# ARIPIPRAZOLE-INDUCED OCULOGYRIC CRISIS (ACUTE DYSTONIA)

**DOI:** 10.1192/j.eurpsy.2023.2143

**Published:** 2023-07-19

**Authors:** L. Rodriguez Andres, C. Vallecillo, L. Gallardo Borge, C. M. Capella Meseguer, G. Guerra Valera, C. Noval Canga

**Affiliations:** 1 Psychiatry; 2 Hospital Clinico Universitario de Valladolid, Valladolid; 3 Complejo Asistencial de Segovia, Segovia; 4 Hospital Universitario de Zamora, Zamora; 5 Hospital Clínico Universitario de Valladolid, Valladolid; 6 Hospital Universitario Central de Asturias, Oviedo, Spain

## Abstract

**Introduction:**

Aripiprazole is a third generation atypical antipsychotic and a dopamine serotonin system stabilizer, effective against positive and negative symptoms of schizophrenia. Within the group of atypical antipsychotics, aripiprazole shows a relatively benign safety profile (e.g. lower metabolic impact, mild effect on cardiovascular parameters), although the reported rate of extrapyramidal side effects is measurable.

Oculogyric crisis (OGC) is a rare movement disorder characterized by a prolongued involuntary upward deviation of the eyes, lasting minutes to hours. In most cases, OCG is a drug-induced adverse event with acute or tardive onset often attributable to a functional impairment of dopaminergic neurotransmission.

**Objectives:**

OGC is seldom reported in children and young adults during treatment with aripiprazole, althouh it is commonly used in youths.

**Methods:**

We report a case of an aripiprazole-induced oculogyric crisis in a 19 year old girl who diagnosed with schizophrenia (paranoid).

**Results:**

There was a complete remission of the OGC’s following aripiprazole dose reduction, suggesting the clinical manifestation was a dose-dependent phenomenon.

**Conclusions:**

The present report should raise awarness among clinicians for this relevant possible adverse event, that can happen also with the use of aripiprazol, not only with typical or more antidopaminergic antipsychotics. Future research in the field should emphasize neurobiological dysfunctions as the basis of EPS/OGC in patients.

**Disclosure of Interest:**

None Declared

